# The effects of an activity-based lifestyle intervention on moderate sleep complaints among older adults: study protocol for a randomized controlled trial

**DOI:** 10.1186/s13063-018-2465-2

**Published:** 2018-01-25

**Authors:** Doris, S.F. Yu, Shamay S.M. Ng, Diana T.F. Lee, Kai Chow Choi, Parco M.F. Siu, Lisa P.L. Low, Jean Woo

**Affiliations:** 1 The Nethersole School of Nursing, The Chinese University of Hong Kong, 6/F, Esther Lee Building, Shatin, NT Hong Kong; 2 Department of Rehabilitation Sciences, the Hong Kong Polytechnic University, ST 506, 5/F, ST Building, No. 8, Shun Yung Street, Kowloon, Hong Kong; 3 The Nethersole School of Nursing, the Chinese University of Hong Kong, Rm 725, Esther Lee Building, Shatin, NT Hong Kong; 4 The Nethersole School of Nursing, the Chinese University of Hong Kong, Rm 722, Esther Lee Building, Shatin, NT Hong Kong; 5 Department of Health Technology and Informatics, Faculty of Health and Social Sciences, Hong Kong Polytechnic University, Room 916, Block Y, Yuk Choi Road, Hung Hom, Kowloon, Hong Kong; 6 School of Health Sciences, Caritas Institute of Higher Education, 18, Chui Ling Road, Tseung Kwan O, NT Hong Kong; 7 Department of Medicine and Therapeutics, the Chinese University of Hong Kong, Prince of Wales Hospital, 9/F, Lui Che Woo Clinical Sciences Building, Shatin, NT Hong Kong

**Keywords:** Sleep complaints, Older adults, Physical activity, Depression, Physical fitness, Mixed method

## Abstract

**Background:**

Moderate sleep complaints are major gerontological issue affecting as many as 80% of older adults. More intriguing findings have indicated that moderate sleep complaints were associated with cognitive decline, functional deterioration, clinical depression, and even morbidity and mortality among older adults. The aim of this study is to evaluate the effects of an activity-based lifestyle intervention on moderate sleep complaint among community-dwelling older adults.

**Methods/Design:**

This sequential quantitative–qualitative mixed method study will randomly allocate 224 individuals to receive either the 16-week group-based moderate-intensity stepping exercise or 16-week health education. The exercise group receives three 60-min stepping exercises per week, whereas the education group receives weekly educative talks on health topics other than sleep. The primary outcomes are sleep quality as measured by the Pittsburgh Sleep Quality Index (PSQI) and sleep pattern as measured by the actiwatch. Physical fitness and mood status are measured as mediating variables by using the Rockport walking test and Profile of Mood States. The qualitative part will invite 30 individuals from the exercise group who have different sleep-related treatment responses to participate in individual interviews to explore their overall perception of using stepping exercise as a lifestyle intervention to improve sleep. Mixed effects model with intention-to-treat analysis will be used for quantitative data. Inductive thematic analysis with a prior coding framework will be used for the qualitative data.

**Discussion:**

By investigating the effects and the mediating mechanism of a moderate-intensity exercise program on moderate sleep complaints among older adults, this study will generate evidence of high scientific value and important public health implication. Understanding the sleep-promoting effects and acceptability of exercise informs how to apply lifestyle promotion as a public health practice to improve late-life moderate sleep complaints and forestall its progression to level of clinical severity.

**Trial registration:**

Clinical Trial Registry Team, Center for Clinical Research and Biostatistics CUHK, CCRB00491. Registered on 1 December 2015.

**Electronic supplementary material:**

The online version of this article (10.1186/s13063-018-2465-2) contains supplementary material, which is available to authorized users.

## Background

### Detrimental health impact of moderate sleep complaint

Sleep problems are a major issue in gerontological care, with as many as 80% of older adults reporting one or more sleep complaints, such as difficulty in falling asleep and nocturnal awakening [1]. Miles (1982) defined a single persistent sleep complaint or the co-occurrence of two or more sleep complaint as a condition called moderate sleep complaints [2]. The prevalence rate of this sleeping problem among older adults were found to be as high as > 40% [3].

It is a common misconception that moderate sleep complaints are an inevitable age-related change and are not counted as a health risk. However, increasingly more compelling evidence has indicated the detrimental effects of moderate sleep complaint in impairing memory and concentration [4], compromising social participation and functional performance, increasing physical symptoms, and increasing fall risk and mood disorders [4, 5]. More intriguing findings have indicated that moderate sleep complaints were associated with an increased risk of clinical depression, chronic diseases including dementia, healthcare utilization, and mortality [4, 6, 7].

## Is exercise an effective lifestyle intervention to promote sleep

An international guideline for health promotion states that intervention targeted at lifestyle factors is the best way to optimize the health of the aging population, as lifestyle intervention is perceived by older adults as more affordable, accessible, manageable, and readily applicable if it is integrated into their daily lives [8]. Exercise has been widely recognized as a lifestyle habit to promote better sleep. Indeed, traditional theories of sleep hypothesized that sleep serves the functions of temperature downregulation of the body and promotes energy conservation and body restoration [9]. Exercise, which elevates the body temperature and creates a metabolic expenditure, therefore triggers a stronger bodily need for sleep. The mood promoting effects of exercise also creates the prerequisite for high-quality sleep [9].

Despite the public recognition and theoretical assumptions on the sleep-promoting effects of exercise, research evidence on this topic is relatively less compelling. Driver and Taylor described this as an “Expectation-Evidence Paradox” [9]. There is a substantial amount of research which examined the sleep-promoting effect of acute exercise. A meta-analysis of 38 studies has identified its modest effect in improving the sleep architecture (e.g. slow-wave sleep) and acute exercise worked even less on improving sleep patterns (e.g. sleeping time, nocturnal awakening, etc.) [10]. It has been concluded that sleep patterns may respond slowly to the bodily metabolic changes. On the other hand, there is compelling evidence to indicate that people with a higher level of physical fitness or athletes reported much better sleep quality and pattern than their less-fit counterparts [9]. Physical fitness, which brings about improved autonomic response and enables higher daytime metabolic consumption, may render the body more readily for temperature downregulation and energy restoration and thereby trigger sleep.

Several pilot-scale studies, indeed, have examined the effects of chronic exercise which improved physical fitness among older adults with moderate sleep complaints. King et al. identified the therapeutic value of a 16-week moderate-intensity endurance exercise program in a randomized controlled trial (RCT) of 43 older adults who had moderate sleep complaints [11], as screened by the Sleep Questionnaire and Assessment of Wakefulness [2]. The program comprised two 40-min group-based training sessions on moderate intensity exercise and two home-based exercise sessions on brisk walking and stationary cycling per week. As compared with a sedentary control after 16 weeks, the exercise arm reported better sleep quality with a 1-h increase in sleep duration and a nearly 50% reduction in sleep-onset latency, after adjusting for their expected treatment credibility. As no sleep-promoting effect was detected at the eighth week of exercise practice, a longer practicing duration might be required to achieve the sleep-promoting effect. Ried et al. reported comparable findings in a sample of 17 sedentary older people and the improved sleep was associated with an improved physical fitness and mood status [12].

Despite such encouraging findings, King et al. failed to identify the sleep-promoting effects of a similar moderate-intensity training program on the objective polysomnographic measure [13]. The non-significant findings might be related to the undue stress caused by the polysomnographic assessment in the sleep laboratory. Another study also failed to identify favorable sleep-promoting effects of exercise of low intensity and shorter duration [14].

The “Expectation-Evidence Paradox” on the sleep-promoting effect of exercise urges for more stringent scientific evaluation of this lifestyle intervention. The positive findings from the pilot studies imply the need for full-scale RCT to examine the effect of moderate-intensity exercise on subjective and objective sleep measures. Future studies also need to avoid using black-box research, but to examine the mediating process between exercise and sleep, so that how and why this lifestyle intervention affects late-life sleep can be elucidated. Indeed, exploring the acceptability and perception of older adults on using exercise as a lifestyle intervention to improve sleep also facilitates the subsequent knowledge transfer. This study aims at addressing all these research agendas.

## Research aims and hypotheses

**Fig. 1:**
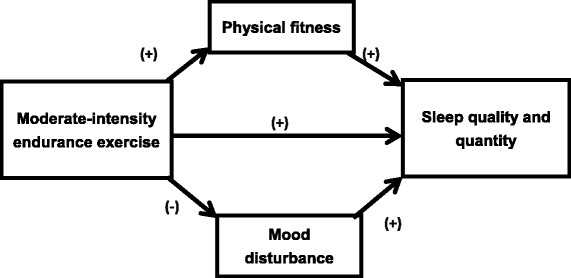
Mediating process of exercise on sleep

The primary aim of this study is to investigate the effects of moderate-intensity endurance exercise training on the sleep pattern and sleep quality of older adults who have moderate sleep complaints. The secondary aims include: (1) examining whether physical fitness and mood status functioned as mediating variables in the effect that the exercise training has on sleep; and (2) exploring the overall perception of older adults in using the exercise program as a lifestyle intervention to improve sleep, particularly the perceived effects, how and why exercise works or does not work, and acceptability, using a qualitative research approach (hence, no hypothesis). Figure 1 outlines the model used to tests mediation for physical fitness and mood status. The research hypotheses to be tested are as follow:

For older adults with moderate sleep complaints,

1. A 16-week moderate-intensity endurance exercise training has greater effect than a non-active attention placebo (i.e. a general health education program) in improving sleep quality and sleep pattern.
2. The effects of the 16-week moderate-intensity endurance exercise training on sleep-related outcomes are mediated by an improved physical fitness and a reduced mood disturbance.

## Methods/Design

### Study design

**Fig. 2:**
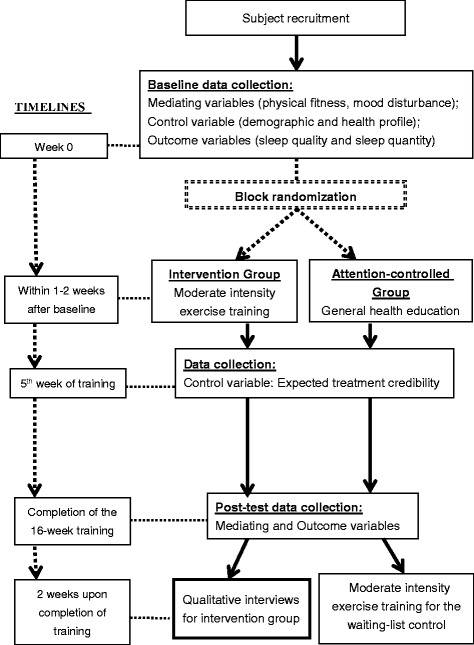
*Flow chart* of study and data collection plan

This is a sequential mixed method study which includes a RCT with a waiting-list attention-controlled intervention to evaluate the effect of a 16-week moderate-intensity exercise training program on sleep quality and sleep patterns and the mediating variables. Older adults with moderate sleep complaints will be randomly allocated to receive either moderate-intensity exercise training or a general education program. The sleep-related outcomes and the hypothesized mediating variables will be measured at baseline before randomization and after completing the study interventions. Then, an individual qualitative interview with a purposive sample of 30 individuals in the exercise arm will be conducted to explore their overall perception of using exercise training for promoting sleep. Particular focus will be placed on how and why they perceive exercise to influence or not influence their sleep and their acceptability of this intervention. The qualitative findings will be used to enhance the interpretation of the quantitative findings about the effect and mediating process of exercise on sleep and to inform the application of the findings. The waiting list control group will receive the exercise training upon completion of the post-test data collection. Figure 2 outlines the overall study design and method.

### Study settings and participants

The study will be conducted in six elderly community centers operated by three large-scale non-government organizations in Hong Kong. Eligible participants will be aged 60 years or above and have moderate sleep complaints, as assessed by the three-item Sleep Questionnaire and Assessment of Wakefulness (SQAW) [2]. The SQAW is a screening tool to identify moderate sleep complaints by assessing the frequency of having difficulties in initiating sleep, maintaining sleep, and early awakening on a “1–5” rating scale in an ascending frequency. A rating of ≥ 3 on any two items or a rating of ≥ 4 on any one item indicates the presence of moderate sleep complaints [2]. Exclusion criteria include participants with: (1) sleep disorder with an organic cause such as sleep apnea; (2) a medical problem (e.g. pain), responsible for the sleep complaint; (3) receiving sleep disorder treatment; (4) cognitive impairment as indicated by an Abbreviated Mental Test score of ≤ 6; (5) engaging in > 60 min per week of moderate or more vigorous exercise in the previous six months; and (6) acute muscular-skeletal problems, stroke, or cardiorespiratory disease including chronic obstructive airway disease and heart failure. A checklist has been developed to screen out the abovementioned exclusion criteria. As this study focuses on moderate sleep complaints, individuals who have reached insomnia of clinical severity according to DSM-V (labelled as insomnia disorder) will be excluded.

Power analysis is used to estimate the sample size estimation for pre-test–post-test study with attrition [15]. According to two pilot-scale studies which examined the effects of moderate-intensity endurance exercise on sleep among older adults [11, 13], the effect sizes of exercise interventions on sleep quality and pattern were in the range of 0.21–1.55 (Cohen’s d). Hence, this study aims to detect a small to medium effect size of 0.4 on the primary sleep pattern and quality outcomes. Using the program RMASS2, it is estimated that a sample size of n = 104 participants per study arm would give the study 80% power at a 5% level of significance to detect the targeted effect size of exercise, assuming randomization will render the two arms equal at baseline and allowing for a 10% attrition. As this study also aims at examining the mediating process between exercise and sleep, consideration is also given to the sample size requirement for path analysis using structural equation modeling approach. According to Loehlin, a sample size of at least 100 and preferable ≥ 200 is required to ensure the statistical power for conducting structural equation modeling [16] and thereby increasing the sample size to 112 individuals per study arm, in allowing for a 10% attrition, to attain the recommended total sample size of 200 participants (i.e. 112 × 0.9 = 100 per arm).

For the qualitative study component, a purposive subsample of 30 older adults who have reported different sleep-related response to exercise training will be invited for individual interviews. This sample size complies with various guidelines which recommend that a sample of 25 participants is adequate to reach data saturation [17, 18] and the sample of qualitative study should lie within 50 [19]. The change in the Chinese version of the Pittsburgh Sleep Quality Index (CPSQI) between the pre-test and post-test period is used as the pre-defined criterion, with ten older adults in each range of 0–35th percentile, > 35th–70th percentile, and > 70th percentile of the score to be selected. The individual selection will also maximize the variation in the socio-demographic background (including age, gender, educational level, and type of housing) within each percentile of the CPSQI score to allow a more comprehensive understanding of the phenomenon. If data saturation has not reached upon the completion of qualitative data collection from the 30 participants, one more case will be recruited from each percentile until no more new findings are detected.

### Study interventions

#### The 16-week moderate-intensity endurance exercise

The moderate-intensity endurance exercise training will be conducted by a research assistant with baccalaureate training in physical education or sport medicine (RA1). The co-authors (NSSM, PS) who are academicians in physiotherapy and exercise physiologist will conduct a three-day training course for the RA1. The exercise training is designed according to the recommendations of the American College of Sports Medicine (ACSM)’s position stand on Exercise and Physical Activity for Older Adults [20]. It is a moderate-intensity 7.5-cm-high bench stepping exercise program for seniors. It is a 16-week program, with three 60-min group training sessions (group size of around ten participants) per week. Each session starts with a 10-min warm-up period using stretching exercise and stationary mobilizing exercise for trunk and limb joints at both upper and lower bodies (e.g. shoulders, elbows, wrists, hips, knees, and ankles) and followed by a session of moderate-intensity endurance exercise. The duration of the endurance exercise will increase gradually from 20 min to the targeted 40 min over four weeks. The endurance exercise includes bench stepping exercise using a 7.5-cm-high stable wooden platform. This exercise has been shown to be very suitable for older people who are less physically active or have reduced joint flexibility and reduced bone strength [21]. Individuals will perform the bench stepping exercise in bouts of at least 10 min each (with a maximum of 5 min of rest in between the exercise bouts) to reach the targeted duration (i.e. 40 min of endurance exercise). Close monitoring and verbal encouragement will be provided by the attending RA1. Moderate-intensity exercise has been widely used among older people and is even safe for older patients with chronic diseases [22]. The Borg Rate of Perceived Exertion (Borg RPE) will be used to monitor the exercise intensity [23]. This is a 15-point scale with scores in the range of 6–20. Descriptors are attached throughout the scale with higher scores indicating more perceived exertion. Physical activity that results in a rating of 12–14 (descriptor: “somewhat hard”) on the Borg RPE is identified as reaching a moderate intensity [23]. The RA1 will explain the Borg RPE scale to the participants and instruct them to speed up or slow down their movements in order to achieve a feeling of “somewhat hard” at the Borg RPE rating of 12–14. The training session will end with a 10-min cool down session with walking exercise and stationary trunk and limb mobilizing exercise involving the joints of the shoulders, elbows, wrists, hips, knees, and ankles. The training will follow the ACSM safety guidelines with assessment done on blood pressure (BP) and heart rate (HR) and contradictory symptoms before training. Attendance rate and adverse events will be recorded.

#### The 16-week health education program

The health education program will be delivered as a non-active attention placebo by a part-time research nurse (RA2). It includes 16 60-min education sessions delivered on a weekly basis. Various health topics on home safety, brain health, foot care, flu prevention, medication management, aging and nutrition, common chronic illness among older adults, voucher scheme for elderly, smart grandparenthood, tips in choosing health screening, etc. will be included. No information relating to sleep and physical activity will be included. Upon the completion of the post-test data collection, the wait-list control group will receive the active study intervention for their sleep problem (i.e. the moderate-intensity endurance exercise program) within the study period.

### Issues of compliance

A previous study examining the effects of exercise therapy among Hong Kong older adults reported high compliance rate of around 80% for these two study interventions [22]. Various strategies, which are based on social cognitive theory and health belief model, will be used to optimize the individuals’ compliance. Table 1 outlines details of these strategies for promoting group cohesion, increasing perceived self-efficacy, and providing emotional and tangible incentives. The first author will provide a one-day training to the RA1 and RA2 on how to incorporate these strategies to the interventions. As the intervener–participant interaction may influence the treatment effect and is difficult to equalize between study arms, a 7-point semantic differential scale will be used to measure the individuals’ perceptions of the characters of the interveners including “friendliness,” “caring,” “sincerity,” “cheerfulness,” and “considerateness” during the post-test data collection. This captures any possible experimenter effect for statistical adjustment if necessary.

**Table 1:** Strategies to optimize compliance to the study interventions

Strategies	Actions
Promoting group cohesion	- Group affiliation: Chinese titles will be assigned to exercise and health education program. Each class and participant will have an arm badge to signify their group identity. - Ice-breaking: Conduct two ice-breaking activities at the first training session to introduce the perception that they are “on the same boat” and have similar sleeping problems. - Goal-setting: The intervener facilitates the group to set the goal of achieving mean attendance rate of not less than 85% for each 2 weeks. The bi-weekly attendance rates will be presented graphically to the group and posted in the training room. - Group socialization: The intervener will foster communication between the participants to increase group socialization at the beginning and at the end of each training session.
Improving perceived self-efficacy	- Effective teaching: A step-by-step approach will be used to equip the individuals with the skills in conducting the intervention. The pace of teaching will be adjusted according to the participants’ learning needs. Clear instruction, demonstration, and return demonstration will be used as core teaching strategies. - Positive evaluation: The interveners will give positive evaluative feedback and encouragement to the individuals in each session.
Emotional and tangible incentives	- Motivational incentives: Three level of certificate awards will be presented to acknowledge the participants with a mean attendance rate of > 90% attendance at the 4th week, 10th week, and 14th week. The awards are titled as Certificate of Good Participation, Certificate of Good Perseverance, Certificate of Active Star, respectively. - Tangible incentives: A souvenir including magnifier, photo frame, coaster, sample hand cream, writing pad, or handkerchief will be provided to the awardees.

### Outcome variables

Sleep pattern and sleep quality are the primary outcomes whereas physical fitness and mood status are the secondary outcomes and mediating variables. The following methods will be used to measure these outcomes at baseline and upon the completion of training. Various potential confounders including demographic background, health profile, and expectation on treatment credibility will also be measured.

#### Sleep pattern

Sleep pattern refers to total sleep time (TST), time to initiate sleep (sleep latency [SL]), number and duration of wakening after sleep onset (WASO), and ratio of TST and the time spent in bed (sleep efficiency [SE]). These parameters will be measured by an actiwatch from the company called ActiGraph, which is a 3.5 × 3.5 × 1 cm watch. It contains an accelerometer to record the intensity and frequency of body movement during sleep. Individuals will wear the actiwatch on the non-dominant wrist during sleep for one-week’s continuous night monitoring. They are required to record the daily in-bed and out-bed time on a simple log book. To retrieve the parameters of sleep pattern, the actiwatch will be connected via a USB port to the ActiTrainer Online Management Platform. With the input of in-bed and out-bed time, an algorithm will translate the raw acceleration data to the sleep parameters. The actiwatch has extensive use in research and is well correlated with the findings on polysomnography [24].

#### Sleep quality

Sleep quality is measured by the 19-item Pittsburgh Sleep Quality Index (Chinese version; CPSQI) [22]. Respondents rate their subjective sleep quality on a “0–3” Likert scale, with higher scores representing poorer sleep quality. The Cronbach’s alpha and test–retest reliability of the CPSQI are 0.82 and 0.77, respectively, with good discriminant validity when used in the Chinese population [25].

#### Physical fitness

Physical fitness is measured by Rockport Fitness Test [26]. This predictive submaximal test requires the participants to walk as fast as possible on a 200-m track for eight rounds during which the individuals can slow down or rest if needed, but instruction to encourage walking will be given. HR will be taken with a HR monitor at the end of every 400 m. A regression equation is used to compute the predicted maximal oxygen uptake (VO_2max_) using the track walk time, fourth quarter HR, body weight, age, and gender. The test–retest reliability is 0.97 and the predicted VO_2max_ demonstrated high correlation (r = 0.93) with the actual VO_2max_ in older adults [26]. Implementation of the test follows the guidelines of the ACSM and BP, HR, and contradictory symptoms will be assessed before the walking test.

#### Mood status

Mood status will be measured by the 30-item Profile of Mood States Short Form (POMS-SF, Chinese version) [27], which covers tension-anxiety, depression-dejection, anger-hostility, vigor-activity, fatigue-inertia, and confusion-bewilderment. A 5-point response scale is used with higher score representing higher mood disturbance. The Cronbach’s alphas were in the range of 0.67–0.90 and the PSOM-SF has been widely used to measure mood disturbance associated with poor sleep.

#### Expectation on treatment credibility

Expectation on treatment credibility refers to the respondent’s expectation of the sleep-promoting effect of the received intervention. A four-item questionnaire is used, which rates the (1) reasonableness of treatment, (2) opinion of the therapist, (3) expectation for improvement, and (4) likelihood to recommend the treatment to others, on a 10-point scale, with a higher score indicating a better expectation [28].

#### Demographic and health profiles

Participants’ demographic and health profile including age, gender, education level, marital status, living conditions, lifestyle, and medical and drug history will be collated.

### Method of randomization and data collection procedure

**Fig. 3:**
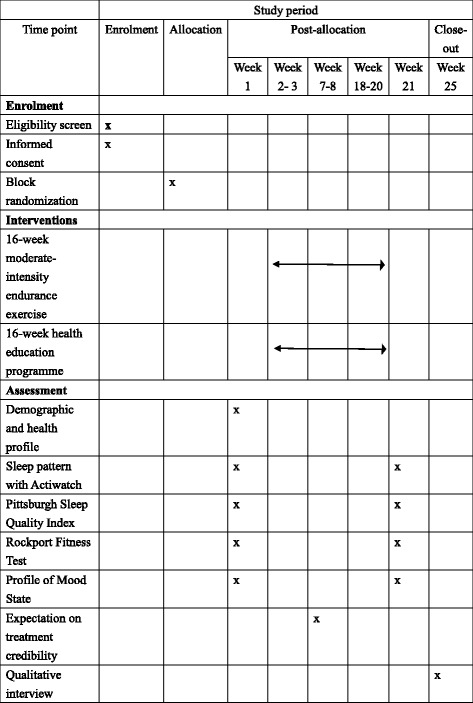
The SPIRIT figure

The social workers in the study settings will help to identify potential individuals for screening. Figure 3 outlines the data collection procedure (SPIRIT figure; SPIRIT 2013 Checklist is appended in the Additional file 1). The RA1 will screen the participants’ eligibility in the community center and obtain their informed consents for participation. Whenever there are about 20 individuals being recruited, s/he will conduct the Rockport Fitness Test to the participants inside the community center and collect data on their demographic and health profile, sleep quality (PSQI), and mood status (POMS-SF). Then, the RA1 will distribute an actiwatch to each individual. S/he will teach them how to: (1) wear and remove the watch for the ten-day recording on sleep pattern (the first three days are for desensitization and data will not be used); and (2) record the in-bed and out-bed time on a simple log book. The part-time research nurse (RA2) will then collect the returned actiwatches after one week in the community center and randomly assign the participants to the study arms.

Block randomization with a block size of about 20 using a restricted shuffled approach will be used to ensure an even distribution of individuals among the study arms. The randomized block is based on the time sequence of participant recruitment. Whenever an individual is being recruited, a consecutively coded number (i.e. participant code number) will be assigned to him/her according to the time sequence of recruitment. After the baseline data collection, the first 20 individuals being recruited (e.g. participant code numbers 1–20) will be evenly randomized to the study arms in a block. A computer-generated random sequence determines the group assignment of these 20 individuals. Twenty paper cards labelled with “exercise” or “control,” with each placed in a single sealed opaque envelop, will be arranged in an order to represent the generated sequence. These envelopes will be numbered consecutively according to this specified order and distributed to the 20 participants according to the participant code sequence. The RA2 will open the envelope for the individuals; with the information on the paper cards represent their group status. The same method will be used to randomize the subsequently recruited participants. Each block of individuals will have an independent computer-generated random sequence to determine the group assignment.

The participants will receive the assigned intervention within two weeks of baseline measure. They will not be informed about whether their received intervention is the tested one. For all individuals, the author (DSFY) will measure perceived treatment credibility at the fifth week of training. This timeframe allows sufficient exposure to treatment before reporting expectations [2]. Another RA (RA3), who is blinded to the participants’ group status, will measure the mediating and outcome variables upon completion of the study intervention for all the study individuals.

After the post-test data collection, the RA2 will interview 30 participants from the exercise arm who have reported different levels of change in the sleep quality. An interview guide with board open-ended questions as outlined in Table 2 will guide the exploration on the individuals’ perception of the exercise training, with particular focus on how and why they perceive exercise therapy would or would not influence sleep and perceived acceptability of this lifestyle intervention. Probes corresponding to the hypothesized mediating variables will be used so that the qualitative findings can enhance the interpretation of the quantitative findings. The interview will be audio-taped to facilitate data analysis.

**Table 2:** Interview guide for the qualitative interview

Questions	Probe
1. How do you feel about the 16-week exercise training?	Acceptability and tolerance
2. How has your sleep quality and pattern changed while you were participating in the 16-week exercise training?	- Sleep latency - Sleep duration - Number and duration of nocturnal awakenings - Early morning awakening - Daytime sleepiness - Feeling refreshed in the morning
3. How is your current sleep pattern and quality different from the situation before you participated in the 16-week exercise training?	
4. You have mentioned that you have improved sleep after participating in the 16-week exercise training. What are the possible reasons leading to such changes? OR You have mentioned that your sleep quality and pattern remain the same after participating in the exercise training. Why is that? What are the possible reasons?	- Physical fitness - Mood change - Perception on sleep disturbance - Perceived effectiveness of exercise training
5. Will you recommend the 16-week exercise training to your friends or family who have disturbed sleep? Why?	
6. Will you consider doing regular moderate-intensity exercise in the future? Why?	

### Data processing and analysis

Data will be double-entered for validation and analyzed on an intention-to-treat (ITT) basis. Skewed variables will be transformed before being subjected to analyses. Baseline characteristics between the two arms of participants will be compared using t test, chi-square test or Fisher’s exact test, as appropriate. Mixed effects models will be used to compare the differential changes on the primary (sleep pattern and quality) and secondary (physical fitness and mood status) outcomes across the baseline and post-test (T2) between the two study arms, with adjustment for the potential control variables (i.e. expected treatment credibility, demographic and health profile) which are statistically non-equivalent at baseline (*p* < 0.25). In particular, a dummy variable (group) will be assigned to represent the exercise training group (i.e. treatment group) with the health education group (i.e. control group) as reference, and another dummy variable (time) will be set to indicate the time points (1 = post-test, 0 = pre-test) in the mixed effects model. To accurately identify the effects of the exercise training, an interaction term group × time will also be included in the model to assess the difference in the change of each outcome across the pre-test and post-test period (i.e. pre-post change) between the two study groups. The statistical significance of the model coefficients, including the interaction term, will be assessed by Wald test. The strengths of mixed effects model lie in accounting for intra-correlation between repeatedly measured data and accommodating missing data caused by dropout, provided the data are missing at random [26]. This method is most suitable for ITT analysis without the need for missing data imputation. All analysis will be conducted by using SAS release 9.3 (SAS Institute Inc, Cary, NC) at a 5% level of significance. Depending on the data distribution, the mixed effects modeling will be performed using the PROC MIXED or NLMIXED.

Path analysis is used to examine the mediating roles of physical fitness and mood status on the effect of exercise training on sleep pattern and quality with adjustment for the above control variables. Based on our hypothesized mediation model (Fig. 1), a baseline path model using the baseline data will be built first. To which path model with the same structure for T2 is added and linked by intra-correlations among the same variables. The final mediation model will then consist of two clusters (corresponding to the two time points) and are linked by the intra-correlations. The equality of a specific path coefficient in the two clusters can be assessed by imposing equality constraint on the coefficient and tested using a nested goodness-of-fit test [27]. The mediation effects of physical fitness and mood status on the effect of exercise training on sleep pattern and quality will be assessed through the significance of the corresponding products of indirect path coefficients from exercise training to sleep pattern and quality, respectively [28]. In view of the possibility of violation of normality assumption, a bootstrapping approach [28], instead of the conventional multivariate delta method, will be used to estimate the standard errors and the confidence intervals of the product terms (mediation effects). Furthermore, the effect of exercise training (as compared to control) on the change of the path coefficients to the sleep pattern and quality outcomes directly or by way through the mediators (physical fitness and mood status) over time can be assessed using the three-way interactions approach [27]. The path analyses and the bootstrapping will be performed using Mplus Version 6 and the parameters will be estimated by mean and variance adjusted weighted least square method (WLSMV). The bootstrapping estimations of the standard errors and confidence intervals will be performed using bias-corrected bootstrapping method with 10,000 replications [28]. The overall fit of the path models is assessed using comparative fit index (CFI), root mean square error of approximation (RMSEA), and standardized root mean square residual (SRMR), with CFI > 0.95, RMSEA < 0.06, and SRMR < 0.08, indicating a good data-model fit [27].

For the qualitative data, the audio-taped interview will be transcribed verbatim by the RA2. The authors will carefully review the transcribed content. Inductive thematic analysis will be used to code the data on the overall perception and perceived acceptability of exercise program. For the findings about why and how exercise influences or not influences sleep, a priori coding framework based on the hypothesized mediating process of exercise on sleep will be used to obtain the data clusters. Inductive thematic analysis will also be used to code the data which does not represented by the model construct. The coded units will be sorted into categories and subcategories and analyzed for recurrent themes and pattern. Within-case and across-case analyses in each group of participants with a similar change in the PSQI score will be conducted and followed by cross-group analysis. Data credibility will be further maintained by conducting an audit trail. The emerged categories will be reviewed for resonance with the quantitative findings. The qualitative findings serve a complementary purpose in concluding the effect, the mediating process, and the acceptability of exercise on sleep among older adults.

## Ethical consideration

Ethical approval for this study has been obtained from the Joint Chinese University of Hong Kong – New Territories East Cluster Clinical Research Ethics Committee (CRE-2010.157-T). The study aims and procedures will be explained to the participants in verbal and through written forms. Written informed consent will be obtained before their participation in the study. Participants will be informed of their right of voluntary participation and they may terminate at any time during the study. Anonymity is fully addressed and participants’ names cannot be identified in any documents and data collection sheets. All the data will only be used for research purpose and will be kept locked in a private place. Only the research team can have access to the data. Approval will be sought from the Ethics Committee before any protocol modification.

## Discussion

Whereas rapid population aging urges effective population policy to tackle the anticipated increase in elderly dependency ratio and reduction in population productivity, strategies to optimize the physical and mental vitality of older adults are crucial to minimize the negative impact of population aging on our society. High-quality sleep is a critical factor to enable older adult to remain active physically, cognitively, and socially, so that they can maintain their social participation and even productivity in their senior years. This study addresses a high prevalent problem of moderate sleep complaints among Chinese older adults. By investigating the effects and the mediating mechanism of a moderate-intensity exercise program on moderate sleep complaints among older adults, this study will generate evidence of high scientific value and important public health implication. For knowledge advancement, findings of this study can clarify the Expectation-Evidence Paradox on the sleep-promoting effect of this well-known lifestyle intervention. As for public health implication, identifying the sleep-promoting effects of the exercise therapy is crucial to inform the treatment options for: (1) improving the sleep quality and pattern of Chinese older adults with moderate sleep complaint; and (2) eventually forestall the progression of moderate sleep complaint to a level where it reaches clinical severity. By focusing on the lifestyle intervention that is acceptable among the older Chinese population, evidence derived from this study will be readily translatable to real-life practice through territory-wide campaign for promoting elders’ health. Findings of this study will be disseminated through local and international conference and publication in international referred journal.

## Trial status

The manuscript reports the protocol for an ongoing trial, for which participant recruitment is currently ongoing.

## Additional file

Additional file 1: SPIRIT 2013 Checklist: Recommended items to address in a clinical trial protocol and related documents*. (DOC 122 kb)

## Abbreviations

ACSM: American College of Sports Medicine
BP: Blood pressure
CFI: Comparative fit index
Co-I: Cooperate Investigator
CPSQI: Pittsburg Sleep Quality Index (Chinese Version)
HKYWCA: The Hong Kong Women’s Christian Association
HR: Heart rate
IST: Total sleep time
PI: Principal investigator
POMS-SF: Profile of Mood States Short Form
RA: Research assistant
RPE: Rate of perceived exertion
SE: Sleep efficiency
SL: Sleep latency
SQAW: Sleep Questionnaire and Assessment of Wakefulness
WASO: Wakening after sleep onset
WLSMV: Variance adjusted weighted least square method
